# Accelerating vaccine trials

**DOI:** 10.2471/BLT.21.020721

**Published:** 2021-07-01

## Abstract

The unprecedented speed with which COVID-19 vaccines have been developed and approved reflects increasingly nimble trial procedures, but opportunities exist for greater collaboration. Gary Humphreys and Lynn Eaton report.

Jeanne Baroni had serious doubts about getting vaccinated. An 81-year-old pensioner, living 40 kilometres outside Paris, France, Baroni was scheduled to walk over to the Melun vaccination centre to receive her first jab of the Pfizer/BioNTech coronavirus disease 2019 (COVID-19) vaccine on 2 February. She didn’t want to go.

“They told me it normally takes years to test a vaccine, but that Pfizer did it in less than a year,” she says. “I thought that it must have been rushed. I had a lot of questions.”

In fact, it takes on average between five and 10 years to develop and approve a new vaccine, clinical trials accounting for the bulk of that time.

Typically split into three phases (although a zero phase is sometimes added), clinical trials comprise a small trial to test safety, followed by a larger trial to test immune response at different doses to select the optimal dose and continue identifying side-effects, and finally a much larger clinical trial to establish efficacy, i.e. whether or not the vaccine actually protects against infection and/or disease.

In the face of the COVID-19 pandemic, and under tremendous pressure from governments worldwide, vaccine developers have had to squeeze these steps into months rather than years.

The clinical trials of the Pfizer/BioNTech vaccine trial took eight months. Trials of the Moderna and AstraZeneca vaccines, the other COVID-19 candidate vaccines approved for use in 2020, were conducted with similar alacrity.

So, they were fast, but were they rushed?

Not according to Dr Marie-Paule Kieny, director of research at the Paris-based Inserm health research institute, and formerly Assistant Director-General for Health Systems and Innovation at the World Health Organization (WHO).

“Increasing the speed of the trials for the leading candidate vaccines did not affect their rigour or the safety of the vaccines ultimately approved,” she says. “The trials were expedited through compression rather than by skipping important stages.”

One way this compression was achieved was to run phases that would normally occur sequentially in parallel. For example, Pfizer and BioNTech started parallel Phase 1 and 2 of trials of their COVID-19 vaccine in late April 2020 and parallel Phase 2 and 3 trials three months later. Other vaccine developers have engaged in similar compressions.

In some instances, pre-clinical (animal) trials and Phase 1 clinical trials have also been run in parallel. This was the case, for example, in the development of both the Moderna and Pfizer vaccines.

“Increasing the speed of the trials […] did not affect their rigour.”

Marie Paule Kieny

Such compressions prompted discussion about where exactly the risk–benefit line should be drawn, notably at the meeting of the International Coalition of Medicines Regulatory Authorities which took place in March 2020 and where most of the participants agreed that data generated in pre-clinical trials should be available prior to enrolling large numbers of human subjects into Phase 2 and 3 clinical trials.

The participants also agreed that the level of testing required depends on the candidate vaccine and the safety profile of the vaccine platform on which it is based.

Vaccine platforms (biotechnology systems that use the same basic components but can be adapted for use against different pathogens by inserting different gene sequences) have come to the fore in the development of COVID-19 vaccines. Messenger ribonucleic acid (mRNA) platforms – the platforms used in the development of the Pfizer and Moderna vaccines – are a case in point.

Such platforms are going to be crucially important in meeting the challenges posed by emerging virus variants. “By introducing a new RNA sequence to code for the expression of a different antigen, such as the famous spike protein which most vaccines are targeting, it is going to be possible to adapt vaccines or make new ones,” explains Dr Marco Cavaleri, the chair of the European Medicines Agency’s (EMA) COVID-19 pandemic task force.

Similarly adaptable (‘adaptive’ in industry jargon) trial protocols will be needed to keep up with what the EMA refers to as variant vaccines.

Developers are already using such protocols in the development of COVID-19 candidate vaccines, notably trial protocols that allow for the testing of different products against a shared control group (the unvaccinated group used for comparison in a trial) and the introduction of new products without requiring new submissions for approval from the trial review board.

Going forward, bridging studies are also likely to figure prominently as developers adapt vaccines to address emerging virus variants. Bridging studies obviate the requirement for large-scale randomized clinical trials by focusing on immunogenicity data (the adapted vaccine’s capacity to provoke an immune response), typically using the quantity of neutralizing antibodies produced as the immune biomarker.

“Where immunogenicity in the candidate replacement vaccine is of the same magnitude as the immune response provoked by the original vaccine, it may be possible to extrapolate efficacy results,” says Cavaleri.

How reliably neutralizing antibodies can serve as a “correlate of protection” (an indication of the protection likely to be provided by a vaccine) is still under discussion. Potential challenges include what is referred to as vaccine-specific immunogenicity – immunogenicity deriving from some factor specific to the vaccine other than the degree to which it provokes neutralizing antibodies.

“Our understanding is still at an early stage,” says Dr Rogério Gaspar, director of WHO’s Regulation and Prequalification Department. “There are plausible scientific arguments that different COVID vaccines might work in different ways. So, we need to be cautious in identifying correlates of protection.”

It is for this reason that WHO recommends making the least changes to the manufacturing processes and platforms in the production of modified vaccines. “This will give us the highest degree of certainty that a modified vaccine will behave in a similar way to the originally authorized vaccine,” Gaspar says.

The trial flexibility on display in COVID-19 vaccine development has not, to date, extended to widespread use of comparative trials, which are going to be needed to answer the questions likely to emerge in the coming months, among them how safely and effectively vaccines can be combined.

“We’re going to need smart solutions to trials as well as smart trial questions.”

Oliver Cornely

“Public health authorities are very interested in getting answers to questions about how well vaccines work together because it will give them more flexibility in their campaigns, but vaccine makers generally less so,” says Cavaleri.

WHO has emphasized the need for collaboration between vaccine developers since the beginning of the pandemic, and encouraged participation in a large international randomized controlled trial known as the Solidarity Trial, the main aims of which were to avoid duplication of effort and to facilitate the simultaneous evaluation of the benefits and risks of different vaccines at multiple sites.

But the trial struggled to attract participants. According to Kieny, one reason for this may have been the focus of the developers, spurred on by governments, on moving fast, and on being first across the finish line as a matter of corporate and national pride.

“Prospective participants [in the Solidarity Trial] were in fierce competition with each other and also working against the clock,” Kieny says. “The WHO protocols for the trial were well conceived, but the willingness of participants just wasn’t there.”

Going forward, different imperatives may emerge. “We are entering a phase where multiple complicated questions about how best to use our resources, including the vaccines we now have, will need answers,” says Professor Oliver Cornely, an infectious disease clinician and clinical trials expert at the University of Cologne and coordinator of the VACCELERATE COVID-19 clinical trial network

VACCELERATE was launched by the European Commission in February 2021, partly to encourage such collaborative trials across multiple countries and to answer the kinds of questions Cornely has in mind.

To date, 16 European Union countries and five associated countries have joined the network which facilitates cooperation and data exchange during clinical trials on COVID-19 therapeutics and vaccines. Just under 400 trial centres have signed up to participate in the network and are making themselves available to researchers wishing to set up studies. As of 31 May 2021, 29 500 individuals had registered to participate in trials organized by the centres.

Among the first fruits of VACCELERATE’s efforts is a small trial (633 people) looking at the immunogenic impact of following up a first dose of the Oxford–AstraZeneca vaccine with the Pfizer vaccine. The study which was led by the Carlos III Health Institute in Madrid, Spain, a VACCELERATE network participant, found that the Pfizer vaccine boosted antibody responses significantly in participants who had received one dose of the AstraZeneca vaccine. A similar study of the Pfizer and AstraZeneca vaccines is being led by the Oxford Vaccine Group using a network of trial sites across the United Kingdom.

Cornely is hopeful that such trials will be the first of many. “It may be true that the first phase of vaccine development has seen a lot of competitive behaviours, but we are also seeing collaboration and how we collaborate in trials is going to be crucial to getting results,” he says. “We’re going to need smart solutions to trials as well as smart trial questions.”

**Figure:**
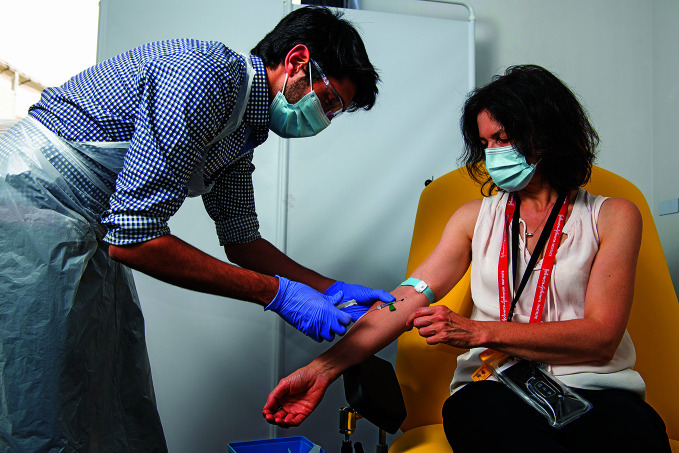
Vaccinating a volunteer in the Phase II Oxford/AstraZeneca vaccine trial at the Centre for Clinical Vaccinology and Tropical Medicine, University of Oxford, United Kingdom of Great Britain and Northern Ireland, June 2020.

University of Oxford/John Cairns

**Figure:**
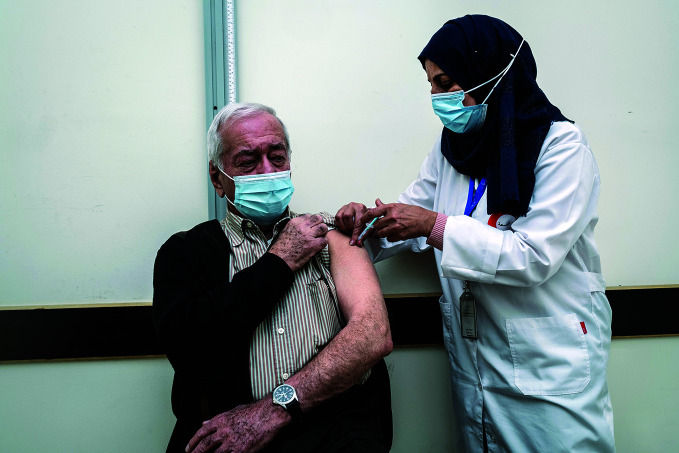
A nurse administers a COVID-19 vaccine at a vaccination site in Ramallah district, occupied Palestinian territory, in March 2021, one year after COVID-19 clinical trials started.

WHO/Noor-Tanya Habjouqa

